# Predicting Sex in White Rhinoceroses: A Statistical Model for Conservation Management

**DOI:** 10.3390/ani13162583

**Published:** 2023-08-10

**Authors:** Leticia Martínez, Paloma Jimena de Andrés, Jose Manuel Caperos, Gema Silván, Jesús Fernández-Morán, Miguel Casares, Belén Crespo, Daniel Vélez, Luis Sanz, Sara Cáceres, Juan Carlos Illera

**Affiliations:** 1 Department of Animal Physiology, Veterinary Faculty, Complutense University of Madrid, 28040 Madrid, Spain; 2 Department of Animal Medicine and Surgery, Veterinary Faculty, Complutense University of Madrid, 28040 Madrid, Spain; 3 Clinical Psychology Unit (UNINPSI), Department of Psychology, Comillas Pontifical University, Calle Mateo Inurria 37, 28036 Madrid, Spain; 4 Zoogical Area of Parques Reunidos Group, Casa de Campo s/n, 28011 Madrid, Spain; 5 Bioparc Valencia, Avenida Pio Baroja 3, 46015 Valencia, Spain; 6 Department of Statistics and Operational Research, Faculty of Mathematics, Complutense University of Madrid, 28040 Madrid, Spain

**Keywords:** reproduction, cortisol, progesterone, estrone, testosterone, steroid hormone metabolites, feces, wildlife, gender determination

## Abstract

**Simple Summary:**

Over the past few years, there has been an increasing interest in comprehending the structure and dynamics of wild populations. This plays a pivotal role in enhancing our knowledge of wildlife management, conservation biology, and behavioral ecology. The primary objective of this study was to construct a mathematical model that could anticipate the sex of the white rhinoceros (*Ceratotherium simum*) by employing non-invasive techniques. The development of this predictive mathematical model—which utilizes concentrations of fecal cortisol, progesterone, estrone, and testosterone metabolites, and which achieves an accuracy rate of approximately 80% for white rhinoceroses—represents a significant and groundbreaking advancement in the realm of wildlife conservation.

**Abstract:**

Ensuring the effective management of every rhinoceros population is crucial for securing a future for the species, especially considering the escalating global threat of poaching and the challenges faced in captive breeding programs for this endangered species. Steroid hormones play pivotal roles in regulating diverse biological processes, making fecal hormonal determinations a valuable non-invasive tool for monitoring adrenal and gonadal endocrinologies and assessing reproductive status, particularly in endangered species. The purpose of this study was to develop a statistical model for predicting the sex of white rhinoceroses using hormonal determinations obtained from a single fecal sample. To achieve this, 562 fecal samples from 15 individuals of the *Ceratotherium simum* species were collected, and enzyme immunoassays were conducted to determine the concentrations of fecal cortisol, progesterone, estrone, and testosterone metabolites. The biological validation of the method provided an impressive accuracy rate of nearly 80% in predicting the sex of hypothetically unknown white rhinoceroses. Implementing this statistical model for sex identification in white rhinoceroses would yield significant benefits, including a better understanding of the structure and dynamics of wild populations. Additionally, it would enhance conservation management efforts aimed at protecting this endangered species. By utilizing this innovative approach, we can contribute to the preservation and long-term survival of white rhinoceros populations.

## 1. Introduction

Poaching pressure is dramatically threatening all the species of rhinoceros worldwide and is heavily damaging wild populations. In the last decade, almost 10,000 rhinoceroses were illegally killed, the majority of them being poached in South Africa. In spite of a slight decrease in the poaching numbers since the peak in 2015, the current poaching rate still stands at up to one rhinoceros killed per day [1]. Increasing anti-poaching efforts and social awareness are trying to ensure a future for the species [2,3], although time is a limiting factor and we urge that appropriate management of every rhinoceros population is maintained [4].

Numerous fields play a crucial role in conservation efforts; nevertheless, recognizing the behavioral traits of a species ought to be regarded as a fundamental element in the formulation of wildlife management and conservation strategies [5]. Additionally, effective management plans play a pivotal role in securing the future conservation of a species [6]. The intricate and diverse range of factors that impact the natural behavior of the white rhinoceros often result in reproductive challenges when kept in captivity; thus, ex situ breeding programs do not produce much success for this species [7]. Therefore, it is necessary to make all possible efforts to improve in situ conservation strategies for the white rhinoceros. Not only is it vital that poaching stops for the long-term survival of the species but it is also necessary to continue expanding populations into their historic range, which can only be achieved by ensuring safe places for their return. For a population to prosper, it must have a suitable habitat and the animals’ behavior must be considered [8]. Adult bull rhinos tend to be solitary and aggressive toward other males. Stress can be the result of aggression or the threat of aggression. Then, an important measure of successful conservation management is achieving adequate sexual distribution in a restricted area, as intraspecific aggression can cause debilitating injuries and even death, and suboptimal sex ratios can exert a detrimental effect on population growth [9]. Taking this into consideration, the identification of individuals’ sex serves as a crucial element within species monitoring programs, aiding in management decisions and enhancing our comprehension of the species’ structure and dynamics [10,11,12,13].

Determining the sex of individuals of many species can be challenging due to the absence of clear sexual dimorphism, particularly in juveniles. Moreover, in elusive or rare species, the task becomes even more complex because direct observation of external sexual gonads is often limited or unavailable. While in the rhinoceros, similar to most mammals, direct observation of the animal might be typically sufficient to differentiate between sexes, it can still pose challenges, particularly when dealing with free-ranging animals in natural habitats.

Several studies have developed non-invasive techniques (which limit animal disturbance) to determine the hormonal profiles in order to monitor and improve the management of different animal populations, especially in endangered species [7,11,14,15,16,17]. In the rhinoceros, the biological samples used to perform non-invasive hormone assays are feces, urine, and saliva [16,18,19]; however, when it comes to wild populations, fecal samples are extensively employed and highly convenient, particularly due to their ease of collection and preservation. Fecal-excreted metabolites are vulnerable to bacterial and environmental degradation, but as long as feces is collected relatively soon after defecation, the metabolites present are a reliable indicator of the physiological state [20]. The stability of fecal metabolites has been shown to last up to several hours after defecation in many species, such as the Asian elephant [21], baboons [22] or different species of avians [23,24].

The reproductive system is a sophisticated endocrine system comprising multiple levels of organization (such as the central nervous system, adrenal glands, tissues, cells, etc.) intricately connected through the hypothalamic–pituitary-gland axis [13]. In addition, in the white rhinoceros, it has been demonstrated to be influenced by other factors, such as environmental factors [25], sociosexual environment [26] or management conditions [16]. Hence, this system exhibits non-linear feedback responses that occur during the interaction among its components and other elements within the body. Fecal glucocorticoids serve as a crucial element in assessing the stress response [27] while also being associated with other factors, including health status [28] and social interactions [16]. Sexual steroid hormones have essential regulatory roles in a wide variety of biological processes, including in behavior, reproduction, sexual dimorphism, development, and brain function, among others [12,29,30,31]. Different concentrations of sexual steroid hormones between males and females deepen our understanding of many physiological mechanisms in relation to the multiple factors influencing the reproductive status of individuals, such as the sociosexual environment, reproductive behavior, or stress conditions, among others [12,26,32,33,34]. While androgens are important precursors of the synthesis of estrogens [32,35], there is evidence to suggest that the ratio of androgens and estrogens could be used as a method to know the sex of an individual of all the species of the animal kingdom [36,37]. Furthermore, the combination of mathematical models and experimental approaches becomes a valuable tool to assess the responses to stimuli made by the endocrine system [38,39]. In addition, molecular studies using DNA testing in feces to determine sex have been also carried out in many wild animals, such as in rhinoceroses [40], elephants [41], wild dogs [42], lemurs [43] or bovids [44], among others. However, this type of study requires a particularly specific and complex methodology in a specialized laboratory. Therefore, the use of a mathematical model that only considers several fecal hormone metabolite concentrations as a tool for sex determination would be an interesting alternative to DNA analysis for those who lack the necessary methodology for molecular testing. 

Numerous contemporary, accredited zoos are actively engaged in ensuring that the species under their care are supported by impactful conservation initiatives that are connected to the survival of those species in their natural habitats. Zoos are conservation resource centers that support conservation efforts outside their facilities. The expertise and methodologies in managing small populations gained through zoological facilities could play a crucial role in supporting the assessment of IUCN Red List species and the development of strategic plans. Additionally, they can aid local governments, national parks, and reserves in formulating long-term management plans and strategies to safeguard depleted and fragmented populations in their natural habitats [45].

Given the importance of determining the sex of individuals in order to gain insights into the structure and dynamics of wild populations, we found it compelling to establish a robust statistical model for predicting rhinoceroses’ sex by using varying levels of fecal steroid hormone metabolites. Thus, our study aimed to develop a mathematical model that utilizes the cortisol, progesterone, estrone, and testosterone metabolites obtained from fecal samples of healthy white rhinoceroses, whether they are housed in captivity or in their natural habitats, in order to predict the sex of unknown rhinoceroses using exclusively single fecal samples. 

## 2. Materials and Methods

### 2.1. Animals, Sample Collection, Conservation and Lyophilization

A total of 562 fecal samples were collected from a total of 15 white rhinoceroses: 7 rhinoceroses housed in two zoological institutions and 8 in a game reserve in South Africa. The details of the animals and sampling are included in Table 1. As the animals were housed in individual enclosures in captivity, fresh fecal samples were intermittently collected from each individual in the morning following defecation over a span of two years. The collected samples were properly labeled and frozen until they underwent further lyophilization. In the free-ranging habitat, samples were obtained by patrolling the reserve prior to daybreak and patiently waiting for fresh feces after locating the rhinoceroses under study. Collecting fecal samples in the wild proved to be significantly more challenging due to the absence of GPS trackers or localization devices. As a result, it was not feasible to collect samples on a strict daily basis for each individual from the game reserve included in the study. Once the sex of the rhinoceros was visually determined, each individual’s sample was collected, precisely identified, and appropriately labeled. Subsequently, it was placed in a portable cooler box and kept in a freezer for later lyophilization. For privacy and safety reasons, the rhinoceroses’ names are coded to avoid identification.

For lyophilization, each fecal sample was homogenized and introduced into the corresponding labeled vial. To freeze-dry the samples, we programmed a Telstar Lyoquest-85 for 48 h at −45 °C under a vacuum pressure of 0.010 mBar. After lyophilization, the samples were stored under vacuum conditions for future analysis. In each sample, the fecal cortisol (COR), progesterone (PRO), estrone (EST) and testosterone (TES) metabolites were measured via competitive enzyme immunoassay (EIA) after an extraction protocol was conducted.

### 2.2. Extraction Protocols

The extraction protocol for the COR and PRO followed a standardized method that was previously published [46,47]. In summary, 0.2 g of dry feces was combined with 0.8 mL of distilled water and 5 mL of methanol. The samples were vigorously mixed for 30 min using a vortex and subsequently centrifuged at 1500× *g* for 10 min. Following centrifugation, 1 mL of the supernatant was transferred to another vial and evaporated under a gaseous nitrogen stream. Subsequently, the samples were reconstituted in 1 mL of methanol for further analyses.

The extraction protocol used for the EST and TES was based on and adapted from the original protocol that was written by Brown and collaborators [13]. In short, 0.2 g of dry feces was combined with 0.5 mL of distilled water and 4.5 mL of ethanol. The mixtures were vortexed for 30 min and centrifuged for 20 min at 1500× *g*. Following centrifugation, 1 mL of the ethanol phase was transferred to a new vial and evaporated at 100 °C. Another 0.5 mL of distilled water and 4.5 mL of ethanol were mixed, and this process was repeated twice. After complete evaporation, the extract was mixed with 3 mL of ethanol and placed in an ultrasonic cleaning bath for 15 min (Sonicator Ultrasons-H, J.P. Selecta, Abrera (Barcelona), Spain). A further complete evaporation in boiling water was then carried out. Finally, the samples were redissolved in 1 mL of methanol and placed back in the ultrasonic cleaning bath for another 15 min. Aliquots were then analyzed via EIA. 

### 2.3. Enzyme Immunoassays 

The quantification of the fecal COR and PRO was performed using a competitive, previously developed EIA [16,47]. In summary, microtiter plates (96-well flat-bottom polystyrene) were initially coated with 250 μL of a 50 μg protein A solution dissolved in 250 mL of coating buffer (0.05 M carbonate/bicarbonate, pH 9.6). The sealed plates were then incubated overnight at room temperature. Subsequently, the plates were washed, and 300 μL of a second coating buffer (Tris PBS-BSA, pH 7.5) was added. Once again, the plates were sealed and incubated overnight at room temperature. The following day, after another wash, 50 μL of EIA buffer (Tris-saline: BSA, pH 7.5) was added to wells A1 and B1 (serving as reaction blanks) as well as wells C1 and D1 (serving as maximum binding wells, or B0). In the subsequent wells, the 7 standards were added in duplicate, ranging from 2 pg/100 μL to 500 pg/100 μL. The other wells of the plate were coated with 50 μL of the test samples in duplicate. Next, 100 μL of the antibody dilution (COR: Cortisol-3 CMO:BSA, Steraloids ref. Q 3889; PRO: P4-3 CMO:BSA, Steraloids ref. Q 2606) was added to all the wells except the blank wells, followed by 100 μL of the biotinylated steroid. Once again, the plates were incubated overnight at 4 °C with agitation. Then, the plates were washed, and subsequently, incubated for 45 min at 4 °C with 250 μL of the conjugate solution (COR: Cortisol-3 CMO: HRP, Steraloids ref. Q 3888; PRO: P4-3 CMO: HRP, Steraloids ref. Q 2605). After washing the plates, a new incubation was carried out under the same conditions with 250 μL of the substrate. This reaction was subsequently halted by adding 50 μL of 20% sulfuric acid. An EIA reader was used to measure the absorbance of the rection, and the hormone concentrations were determined using specialized software (Bio-Tek Instruments ELx808, Alemania) and reported in ng/g of feces. 

The quantification of the fecal EST and TES levels was performed using a competitive EIA that was developed and validated at the Endocrinology Laboratory of the Department of Animal Physiology, Veterinary Faculty, Complutense University of Madrid [15,48]. In summary, similar microtiter plates were coated overnight at 4 °C with the proper antibody dilutions (EST: E1-3-Gluc-BSA, Steraloids ref. E 2320; TES: Testosterone-3 CMO-BSA, Steraloids ref. A 6958). Following that, the plates were washed three times and conjugate working solutions (EST: E1-3-Gluc-HRP, Steraloids ref.; TES: Testosterone-3 CMO: HRP, Steraloids ref. A 6957) were prepared by diluting the conjugate stocks in assay buffer. All the standards and extracted samples were diluted in conjugate working solutions and analyzed in duplicate. To induce a competitive reaction, the plates were incubated at room temperature for 2 h. Subsequently, the plates were washed three times with wash buffer, and Enhance K-Blue TMB substrate (Neogen, Lexington, KT, USA) was added to every well. A stop solution was added to halt the colorimetric reaction, and the absorbance was measured at 450 nm using an automatic microplate reader. The hormone concentrations were calculated using dedicated software (ELISA AID 1.0, Eurogenetics, Seraing, Belgium) specifically developed for this technique. A standard dose–response curve was generated by plotting the binding percentage (B/B0 × 100) against the concentrations of the steroid hormone standards. The concentrations of EST and TES were also expressed in ng/g. The validation parameters of the technique, including the sensitivity, recovery rates, and intra- and inter-assay coefficients of variation, as well as parallelism, were assessed following the methodology previously reported by Illera et al. [49].

*Validation parameters.* The EIA technique’s sensitivity was assessed by determining the low detection limit and calculating it over 10 consecutive assays. The sensitivity results were COR = 0.14 nmol/L, PRO = 0.25 nmol/L, EST = 0.31 nmol/L and TES = 0.13 nmol/L. The EIA technique’s accuracy was assessed by determining the recovery rates of the known amounts of COR, PRO, EST, and TES, and these were spiked into fecal samples. The average range of the recovery rates, presented as the mean ± standard deviation, was as follows: COR = 94.29 ± 3.85 to 97.15 ± 2.68; PRO = 94.29 ± 3.85 to 98.71 ± 5.37; EST = 90.08 ± 3.29 to 93.27 ± 0.90; and TES = 90.75 ± 3.92 to 99.65 ± 2.01. The precision of the COR, PRO, EST, and TES was evaluated by calculating the coefficients of variation (CV %) for both the intra-assay and inter-assay measurements. The coefficient of variation within the assay (intra-assay CV) was determined by performing replicate measurements of three standard concentrations of the hormone (20 pg/mL, 200 pg/mL, and 2 ng/mL) and ten samples. Each sample was tested twice, for a total of 10 times within a single assay. The coefficient of variation between the assays (inter-assay CV) was calculated by performing replicate measurements of the same samples mentioned above in 10 consecutive assays. The results for the CV were as follows: COR = intra-assay: 14.0 ± 1.02%, inter-assay: 9.1 ± 1.23%; PRO = intra-assay: 3.2 ± 0.98%, inter-assay: 5.3 ± 0.89%; EST = intra-assay: 2.9 ± 0.84%, inter-assay: 6.5 ± 1.36%; and TES = intra-assay: 6.7 ± 1.32%, inter-assay: 9.1 ± 2.14%.

To assess the impact of fecal material on the standard curve, parallel standard curves were constructed using samples containing fecal material alongside the standard dose–response curve. A high degree of parallelism was observed between the standard curves for the hormones under investigation: COR, R = 0.84; PRO, R = 0.87; EST, R = 0.83; and TES, R = 0.89. Table 2 illustrates the cross-reactivity percentage of the polyclonal antibodies toward the closely related steroids.

### 2.4. Statistical Analyses

The level of the fecal steroid hormones was expressed as the continuous variables, hereinafter referred to as the COR, PRO, EST and TES. The mean value and the mean standard error were employed for the descriptive analysis of the continuous variables. The results were expressed in ng/g of dry feces. The variable SEX was used to identify the sex of the animal, with a value of 0 for males and 1 for females. Taking into account that the hormones tested do not have a normal distribution, the Kruskal–Wallis test was used instead of an ANOVA test to measure the degree of association of the sex variable with these hormones. Pearson’s correlation test was utilized to assess the level of association between the tested hormones.

Starting from these data, a statistical model—based on the use of a logistic regression model—was designed to relate the sex of a rhinoceros with the levels of COR, PRO, EST and TES obtained from fecal samples. The following formula was used in the logistic regression model:

$$P\left(SEX=1\right)=\frac{1}{1+e^{\beta_{0}+\beta_{1}*COR+\beta_{2}*EST{+\beta }_{3}*PRO+\beta_{4}*TES+\varepsilon }}$$

For this purpose, SAS v9.4 software was used for the data processing and, specifically, the LOGISTIC procedure of the STAT module for the model fitting.

### 2.5. Description of the Statistical Method Proposed

The statistical model was adjusted in order to predict the sex based on the fecal hormonal levels using only one fecal sample. Firstly, the formula was applied to all the collected fecal samples, with the aim of providing a methodological description and analyzing the inclusion of different fecal hormones in the model. Secondly, to perform a biological validation of the method, various regression models were created excluding all the samples from a particular animal in each model. Subsequently, the accuracy of the method for sex determination was analyzed by applying the hormonal results of each of these excluded fecal samples (which were hypothetically of unknown sex) in the created regression models in order to obtain the predictive value of the method.

Therefore, a total of 562 observations were included, with the dependent variable being the “sex” of the animal and the independent or regressor variables being the values associated with each of the hormones mentioned. This model provides, for each fecal sample from each rhinoceros, the probability that it comes from a female. If this probability is greater than 0.5, the rhinoceros is considered female; otherwise, it is considered male. 

*Validation of the model.* Since it was challenging to obtain a high number of animals for the study, a total of 15 logistic regression models were fitted. In each model, the fecal samples from a different rhinoceros were excluded, and these excluded samples were employed for the validation of each respective model. Through each of the models, the sex of the animal on which the model was not trained was predicted by estimating it using a system analogous to the one mentioned above. Later on, a different inclusion of variables was selected to test the predictive models.

## 3. Results

### 3.1. Steroid Hormones

**Figure 1:**
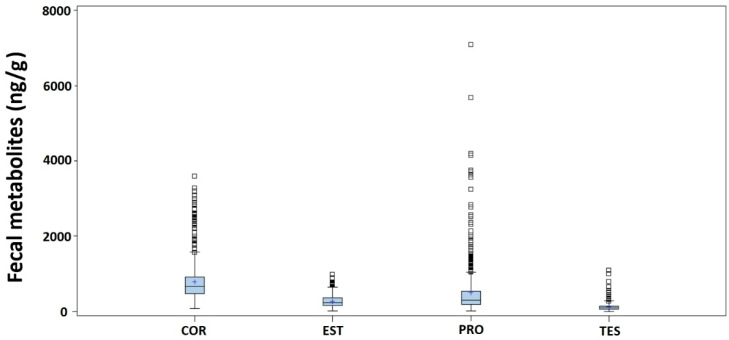
Box and whisker plots for the fecal cortisol (COR), estrone (EST), progesterone (PRO), and testosterone (TES) metabolites (ng/g of dry feces) measured in the 15 animals included in the study.

The estimated mean values of the four hormone metabolites, together with their standard errors and the 95% confidence intervals for the population means, are shown in Table 3. Figure 1 shows the box and whisker plots of the data collected for the four hormone metabolites, showing in all cases the high presence of outliers (which are somewhat more limited in the EST).

**Figure 2:**
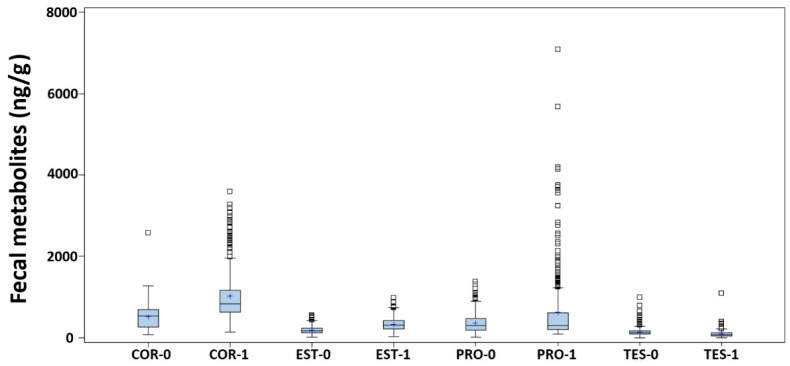
Box and whisker plots for the fecal cortisol (COR), estrone (EST), progesterone (PRO) and testosterone (TES) metabolites (ng/g of dry feces) measured in the 15 animals (males = 0, females = 1) included in the study.

Table 4 shows the mean estimates of each hormone for males and females. The means of the COR, PRO, and EST were significantly higher in females, while the estimated mean value of the TES was significantly higher in males. The box and whisker plots for the data distributions associated with the variables by the sex of the animal are shown in Figure 2.

### 3.2. Association between the Sex and the Hormones Assayed and Among the Hormones

The COR and EST are the hormones that show the clearest differences between the male and female rhinoceroses. However, as displayed in Table 5, there are significant differences between the sexes in all the hormones studied. 

Finally, in Table 6, it can be observed that the hormonal variables can be considered uncorrelated—except for the EST, which is significantly correlated with the COR (*p* < 0.001) and PRO (*p* < 0.001).

### 3.3. Sex Determination Using the Statistical Model

At first, an initial logistic regression model was fitted to the 562 records associated with the rhinoceroses’ fecal samples using a stepwise variable selection process. Table 7 shows the order in which the variables have entered the model, and it can be observed that none of the proposed variables have been excluded from the model.

Table 8 shows the estimates, standard errors and odds ratios associated with each of the regressor variables in the model. It can be observed that the TES is the only hormonal indicator with a negative sign (equivalently, its odds ratio is less than 1), such that the identification of high levels of this hormone in an animal’s feces increases the probability that it comes from a male. Conversely, high levels associated with any of the other hormones increase the probability that they come from a female, with the EST increasing this probability the most (odds ratio = 1.008), followed by the COR (odds ratio = 1.004) and PRO (odds ratio = 1.001). 

*Validation of the model.* In order to perform a biological validation of the method, fifteen logistic regression models were adjusted using the four mentioned variables. In each one of the models, the samples associated with each one of the rhinoceroses were excluded, being used then to test the accuracy rate of the model. It was observed once again that all the variables that were involved maintained the sign of the parameter estimate and, therefore, the underlying logic under them (Table 9). In addition, only in one of the fifteen models (model 4), one of the variables (PRO) showed a *p*-value higher than 0.05. 

The obtained models were highly valuable in terms of the predictive performance: the sex of the hypothetically unknown animal was correctly predicted in 447 fecal samples excluded from the model training process, resulting in a methodology accuracy rate of 79.54% (Table 10).

Furthermore, considering that this type of model is influenced by the presence of outliers, as observed in Section 3.1, the results obtained were contrasted with those that would be obtained by applying logarithmic transformations to the hormonal variables to dampen their influence. However, since these transformations did not provide improvements from the point of view of the predictive results obtained, and as their consideration did not allow a direct interpretation of the odds ratios from the hormone levels but from their logarithm, it was decided not to apply these transformations.

Subsequently, it became intriguing to examine the impact of the formation of different groupings of hormone metabolites on the predictive value of the model. To this end, a more restrictive selection of variables was performed to predict the sex of the animals using the excluded fecal samples, which were assumed to be of unknown sex. When grouping the TES and EST, or when grouping the TES, EST, and PRO, similar results were observed. In both cases, the accuracy rates of the methodologies were close to 70% (Table 11), noticeably lower than the one obtained with all four studied hormones, which was approximately 80% (Table 10).

## 4. Discussion

Determining the sex of individuals plays a crucial role in species monitoring programs, offering valuable insights for management decisions and enhancing our comprehension of species’ structure and dynamics. Ensuring an appropriate sexual distribution within a confined area becomes a significant factor in effective conservation management. This measure is crucial, as intraspecific aggression can lead to severe injuries and fatalities, while suboptimal sex ratios can adversely affect population growth [9,10,11]. 

Some authors have found evidence supporting the notion that reproductive hormone levels determine the gonadal sex in all the species of the kingdom animal [36,37]. Hence, in this investigation, we quantified the concentrations of various fecal hormone metabolites (cortisol, progesterone, estrone, and testosterone) with the aim of establishing, for the first time, a predictive statistical method for determining the gonadal sex in the white rhinoceros. Our objective was to integrate mathematical modeling and experimental approaches to evaluate the responses of the endocrine system. While it is typically feasible to differentiate between the sexes of rhinoceroses through direct observation, there are instances where accurately identifying individuals becomes challenging. This complexity arises when, for instance, visual confirmation of the animal is not possible and we are limited to analyzing its biological remnants, such as feces. Therefore, we deemed it valuable to develop a model that enables sex identification through assessing the relative concentrations of specific fecal steroid hormone metabolites. 

The use of non-invasive methods for hormonal determinations is gaining special importance in studies of the physiology, animal welfare, and behavior of threatened species, specifically those with the aim of contributing to their conservation [14,33]. This sampling method for measuring stress and reproductive hormones offers several benefits, as it can be conducted without the requirement for physical or chemical restraint. Such restraint methods often induce unnecessary stress in the animals and may potentially affect the accuracy of the hormone measurements [50]. Moreover, the efficiency and cost-effectiveness of the sampling make this technique suitable for long-term studies and repeated sampling. In addition, it allows samples to be obtained without being in direct contact with the animal, avoiding the risks that this entails for handlers and wildlife animals. Furthermore, since the stability of glucocorticoids when in feces for several hours being subjected to different humidity and temperature conditions has already been established [21,22,23,25], this methodology opens up a wide range of applications in nature, where it is sometimes a difficult task to obtain recently deposited stool. Indeed, the utilization of fecal sampling is highly regarded as a valuable approach for studying various hormones in wild species [17,47,51,52,53].

Considering all these factors, we conducted an analysis of the steroid hormone metabolites in fecal samples, aiming to predict the gonadal sex. The performance characteristics (e.g., parallelism, recovery rates, sensitivity, precision) of these assays were verified as accurate, precise, and having an appropriate range of sensitivity. In our study, we observed that the average concentrations of the COR, PRO and EST were significantly higher in females. On the other hand, the estimated mean value of the TES was significantly higher in males, which aligns with our expectations. Interestingly, the COR and EST exhibited the strongest correlation with sex, making them the most significant hormones in the model we developed. This could be explained by the fact that the EST was the hormone with the fewest outliers detected and with a better correlation with sex, as determined by the Kruskal–Wallis test. In addition, the PRO and TES were also significantly correlated with sex. The crucial event in mammalian sexual differentiation occurs in the embryonic stage of sex determination, when the bipotential gonads differentiate as either testes or ovaries according to the sex chromosome constitution of the embryo, XY or XX, respectively. Once differentiated, the testicles produce sexual hormones that induce the subsequent differentiation of the male reproductive tract. On the other hand, the lack of masculinizing hormones allows the formation of the female reproductive tract in XX embryos. Later on, in the sexually mature individual, the gonads (testes or ovaries, as appropriate) are the main organ for the synthesis of sex hormones to regulate sexual characteristics as well as the reproduction and sexual behavior in each sex under the control of the hypothalamic–pituitary axis [32,54]. Therefore, our results are consistent with the normal functioning of the reproductive system and are valid as a method of internal validation for the EIA technique. Nonetheless, achieving normal hormonal values in each individual is dependent on the appropriate functioning and interplay of steroidogenic tissues, as well as on the involvement of enzymes in their synthesis and transport. These factors can be influenced by various elements, such as the seasonal androgen variation [25], territorial behaviors [55], sociosexual environments [16,26,34] or reproductive cycles [56]. 

In the white rhinoceros, the role of the different steroid hormones has been previously studied [7,16,25,26,34], although there is a paucity of data regarding the interrelation of estrogens, progestogens, androgens and glucocorticoids in this species. Based on the findings of this study, the hormonal variables assessed can be considered uncorrelated—except for the EST, which was significantly correlated with the COR. A similar unexpected finding has been documented in spotted owls, where the fecal samples exhibiting elevated levels of fecal estrogen metabolites were also found to contain high concentrations of fecal glucocorticoids. The mechanisms by which sex steroids modulate glucocorticoid feedback on the hypothalamic–pituitary–corticotrope axis in rhinoceroses remain elusive. However, in humans, it has been observed that sex steroids influence specific gender-related adaptations of the hypothalamic–pituitary–adrenal axis in response to cortisol feedback. This correlation highlights the interconnectedness within the endocrine system and emphasizes the need for comprehensive investigation in the rhinoceros species. 

Taking all of these factors into account, we proposed a mathematical method based on the levels of fecal hormone metabolites to predict the sex of unknown rhinoceroses using solely single fecal samples. The method is a logistic regression model that establishes the probability that a certain fecal sample came from a female. Subsequently, a biological validation of the model was performed to test its predictive value using fecal samples from animals of hypothetically unknown gender. This prediction was performed independently for each fecal sample. By following this procedure for each validation dataset, we have determined that the sex of the rhinoceros is accurately predicted based on a single fecal sample in 79.54% of cases. If the model only considers the variables EST and TES, this rate decreases to 69.75%, while including all the hormones except COR yields a rate of 68.68%. Therefore, the most favorable results were obtained when all four hormones studied were incorporated into the predictive model. The proposed validation method is based on excluding the fecal samples from a specific animal and treating them as samples from hypothetically unknown individuals, thereby testing the accuracy of the model. This validation method could have been employed from the beginning, although the inclusion of a larger number of samples leads to better predictive results. In most studies involving wild animals, the number of animals always poses a limitation [15,16,17], unlike predictive statistical models based on hormone values in humans, where a much larger number of samples can be used for the method design and validation due to their easy availability [39]. However, the utilization of 562 samples for developing our method appears to be sufficient for obtaining reliable prediction results. Previous studies have tried to develop sexing methods for wild species. However, these studies are only based on different hormone ratios. Bautista and collaborators [12] established a sexing method for great bustards based on the basic rule that the total testosterone concentration should be higher than the total estradiol from individual males, and the opposite should occur in females. Their findings accurately sexed the different individuals in their study. In agreement with these authors, our results showed higher mean testosterone metabolite concentrations in males and higher mean estrogen metabolite levels in females; however, this result did not occur in every study sample. Taking into account only these two hormones, the method yielded inaccurate predictions in over 30% of the samples. When additional hormones were incorporated into the mathematical method, the inaccurate predictions decreased to as low as 20%, highlighting the importance of considering other hormones to enhance the outcomes of this methodology. Interestingly, another study on spotted owls noted that the ratio of estrogen to testosterone was mainly influenced by the reproductive and physiological condition of the animal [23], as may also be the case in our study. According to these authors, our results, which showed great variability between the different individuals, could be influenced by the reproductive and physiological state of the animals. This is because the older males and females had a higher percentage of hits, and we found a worse adjustment in the younger individuals or supposedly pregnant females. Since androgens are precursors of estrogens [35,54], if the estrogen concentration rises, the precursor decreases. Females’ reproductive cycles vary estrogen production periodically, and during pregnancy, the estrogen and androgen concentrations are maintained at baseline levels [32,35,47,56], which may also influence the hormonal results for sex determination. This could be the reason why the best data in the mathematical models are obtained when all the hormones of the study are taken into account to design the predictive method, since it was intended to minimize prediction failures and to better reflect the effects of external factors on the endocrine axis, which is achieved through the combination of the different hormones studied. However, and as a note to our methods, the three rhinoceroses for which the sex estimation failed with the second method were the same as those misclassified by the first method even when including all four hormone metabolites. Still, there should be many factors that must be considered when determining the sex based on hormonal analysis, as it has been previously mentioned.

On the other hand, genetic methods have also been employed to determine the sex in various animal species [40,41,42,43,44]. However, there are several limitations to conducting DNA assays in fecal samples for determining sex. These include DNA degradation caused by exposure to environmental elements like heat, moisture, and microbial activity, which can impact the quality and quantity of extracted DNA, making it difficult to obtain reliable results. Additionally, contamination is a concern, as fecal samples may contain DNA from various sources, such as microorganisms, prey species, or other individuals. Furthermore, fecal samples generally have lower DNA concentrations compared to other sample types, such as blood or tissue. The presence of polymerase chain reaction inhibitors in feces can also interfere with DNA amplification, limiting the reliability and sensitivity of sex determination assays or introducing variability in marker sequences [57,58,59]. According to previous estimates, the cost of DNA testing for sex determination is approximately USD 1 per sample, excluding labor costs and only considering the expenses for reagents and plates [41]. The estimation of the costs for the four hormonal determinations per fecal sample is around USD 0.8 in our laboratory. The motivation for offering an alternative method is not solely based on lower costs but also on the interest in providing an alternative approach. Hence, we present a cost-effective alternative to the current genetic analyses, thereby facilitating population management in the wild. Consequently, this type of analysis holds significance not only for assessing the species under study but also for conducting investigations in other wild species.

The white rhinoceros is listed as a near-threatened species, and numerous conservation strategies, including captive breeding programs with the purpose of reintroducing individuals into the wild and repopulating traditional habitats, have been designed. These captive environments are an invaluable resource that provide many advantages regarding sample management and the monitoring of health status, which enable the collection of sufficient and objective information. The advantages of using samples from these institutions are the following: the guarantee of using healthy animals and the availability of sufficient samples to establish a starting point for many researchers. Thus, the white rhinoceroses used for this study had complete and thoroughly monitored clinical histories, and these were more than sufficient to provide valuable samples for this study. 

## 5. Conclusions

In conclusion, our study represents a significant step in integrating mathematical modeling and experimental approaches to evaluate the endocrine system’s responses in white rhinoceroses. By quantifying the concentrations of the fecal hormone metabolites, we have successfully developed a predictive method for determining the sex of unknown rhinoceroses using single fecal samples. The inclusion of multiple hormone metabolites, including cortisol, progesterone, estrone, and testosterone, has proven crucial for achieving accurate and reliable predictions. Our non-invasive sampling method offers numerous advantages, such as reduced stress in the animals, cost-effectiveness, and the ability to conduct long-term studies. The findings from our study contribute to the understanding of sex determination in white rhinoceroses. We observed significant differences in the hormone concentrations between males and females, with the cortisol and estrone metabolites exhibiting the strongest correlation with sex. These results highlight the complex interplay within the endocrine system and emphasize the need for comprehensive investigations in the rhinoceros species. Furthermore, our proposed mathematical method for predicting sex based on fecal hormone metabolites shows promise for application in population management and conservation efforts. The reliability and accuracy of our method, achieving a prediction rate of 79.54% when incorporating all four hormone metabolites studied, make it a valuable tool for assessing the sexual composition of rhinoceros populations. This information is crucial for effective conservation management, ensuring an appropriate sexual distribution within confined areas and mitigating the risks of intraspecific aggression and suboptimal sex ratios.

## Figures and Tables

**Table 1:** Details of the origin of the animal, sex, age (years) and the number of samples collected from the 15 white rhinoceroses included in the study.

Origin of Rhinoceros	Sex	Age at Sampling	Number of Samples
Zoo Madrid (N = 3)	Male	40	34
	Female	15	46
	Female	40	46
Bioparc Valencia (N = 4)	Male	20	83
	Male	35	70
	Female	6	62
	Female	7	69
South African Reserve (N = 8)	Male	7	26
	Male	7	27
	Male	9	22
	Female	10	21
	Female	13	15
	Female	21	16
	Female	30	13
	Female	30	12
Total (N = 15)	Males (N = 6) Females (N = 9)	19.7 ± 6.01 * 19.1 ± 3.97 *	N = 262 N = 304

* Mean age ± standard error of the mean.

**Table 2:** Percentage of cross-reactivity of polyclonal antibodies against related steroids.

Steroid Compound	PRO	TES	SUL	COR
Testosterone	0.48	100.00		
Testosterone 3-carboxymethyloxime		100.00		
Androstenedione 3-carboxymethyloxime: BSA				
5α-dihydrotestosterone		20.00		
5β-dihydrotestosterone		5.00		
Androstenedione		6.12		
Androstenediol		3.50		
Androstenolone		3.21		
Epitestosterone		0.10		
Estrone	0.01	0.01	100.00	
Estriol	0.01	0.01	0.20	
17β-estradiol	0.01	0.01	11.70	
Progesterone	100.00	3.74	0.20	
Cortisol	0.01	0.05	0.20	
Androstenedione 3-carboxymethyloxime: BSA				
5α-hydroxyandrosterone				
11-deoxycortisol		0.01		
6β-hydroxyandrostenedione				
11β-hydroxyandrostenedione				
Dihydroepiandrosterone				
6 keto 17β-estradiol carboxymethyloxime				
16 keto 17β-estradiol				
6 keto 17β-estradiol				
2 hydroxyestradiol				
6 hydroxyestradiol				
17β-estradiol benzoate				
17α-estradiol				
Progesterone				
11α hydroxyprogesterone hemisuccinate	110.00			
11α hydroxyprogesterone	14.03			
17α hydroxyprogesterone	0.01			
20α hydroxyprogesterone	0.01			
20β hydroxyprogesterone	0.01			
Pregnenolone	21.73			
Estrone-3-sulfate			100.00	
Estrone-3-glucuronide			200.00	
Estradiol-3-glucuronide			5.70	
Estradiol-3-sulfate			5.00	
Estradiol-17-sulfate			0.20	
5α-pregnan-3β, 11β, 21-triol-20-one				100
5α-pregnan-3β, 11β, 20β, 21-tetrol				110
5α-pregnan-3β, 11β, 17α, 21-tetrol-20-one				45
5α-androstan-3β, 11β-diol-17-one				230
Cortisol				0.80
Corticosterone				0.73

**Table 3:** Estimated means, mean standard errors, standard deviations, and 95% confidence intervals for the population mean of the four hormone metabolites (ng/g of dry feces).

	N	Mean (ng/g of Dry Feces)		Standard Deviation	95% CI for the Mean	
		Estimation	Standard Error	Estimation	Low Limit	Upper Limit
COR	562	791.32	23.809	564.440	744.554	838.087
PRO	562	502.20	28.068	665.394	447.070	557.332
TES	562	117.98	4.371	103.630	109.393	126.565
EST	562	264.10	6.505	154.220	251.322	276.878

**Table 4:** Estimated mean and standard error for each hormone by sex (ng/g of dry feces). Standard deviations and 95% confidence intervals for the population mean by sex (0 = males, 1 = female).

SEX		N	Mean (ng/g of Dry Feces)			Standard Deviation	95% CI for the Mean	
			Estimation	Standard Error	*p*-Value	Estimation	Low Limit	Upper Limit
0	COR	262	522.893	16.969	<0.0001	274.666	489.480	556.306
	PRO	262	358.921	15.274	<0.0001	247.227	328.846	388.996
	TES	262	148.031	7.217	<0.0001	116.816	133.821	162.242
	EST	262	187.837	6.208	<0.0001	100.477	175.614	200.059
1	COR	300	1025.747	37.132	<0.0001	643.140	952.675	1098.818
	PRO	300	627.333	49.788	<0.0001	862.356	529.355	725.311
	TES	300	91.733	4.745	<0.0001	82.190	82.395	101.071
	EST	300	330.704	9.357	<0.0001	162.063	312.290	349.117

**Table 5:** Kruskal–Wallis test between the variable sex and the four steroid hormones (N = 562).

		COR	PRO	TES	EST
SEX	Chi-square	147.8827	6.1364	69.0615	125.9113
	*p*-value	<0.0001	0.0132	<0.0001	<0.0001

**Table 6:** Pearson’s correlations between the four steroid hormones (N = 562).

		COR	PRO	TES	EST
COR	Pearson’s correlation	1	−0.036	−0.068	0.272 **
	Sig. (bilateral)		0.391	0.108	0.000
PRO	Pearson’s correlation	−0.036	1	−0.016	0.128 **
	Sig. (bilateral)	0.391		0.706	0.002
TES	Pearson’s correlation	−0.068	−0.016	1	−0.060
	Sig. (bilateral)	0.108	0.706		0.153
EST	Pearson’s correlation	0.272 **	0.128 **	−0.060	1
	Sig. (bilateral)	0.000	0.002	0.153	

** *p* < 0.001 (bilateral).

**Table 7:** Summary of the hormone inclusion in the stepway process.

Step-by-Step Selection Summary					
Step	Effect		DF ^a^	Chi-Squared	Pr > ChiSq
	Introduced	Deleted			
1	EST		1	120.2383	<0.0001
2	COR		1	81.6622	<0.0001
3	TES		1	46.6582	<0.0001
4	PRO		1	17.3752	<0.0001

^a^ DF = Degrees of freedom.

**Table 8:** The parameter estimates and odds ratios of the method that includes all four hormone metabolites assayed.

Parameter Estimates and Odds Ratios (TES, EST, PRO and COR)								
Parameter Estimates						Odds Ratio Estimates		
Parameter	DF	Estimation	Error	Chi-Squared	Pr > ChiSq	Point Estimate	95% Wald Confidence Interval	
Intercept	1	−3.6273	0.4337	69.9442	<0.0001			
COR	1	0.00381	0.00044	73.9753	<0.0001	1.004	1.003	1.005
PRO	1	0.00124	0.00034	12.9621	0.0003	1.001	1.001	1.002
TES	1	−0.0114	0.00193	35.217	<0.0001	0.989	0.985	0.992
EST	1	0.00768	0.00102	56.2822	<0.0001	1.008	1.006	1.010

**Table 9:** Parameter estimates and standard deviations for each of the 15 fitted models that consider the 4 hormone metabolites.

Model	Parameter	Intercept	COR	PRO	TES	EST
1	Estimate	−3.9145	0.00267	0.00147	−0.00814	0.00909
	Standard Error	0.4577	0.000444	0.000359	0.00193	0.00113
2	Estimate	−3.6034	0.00375	0.00122	−0.0114	0.00769
	Standard Error	0.4343	0.000448	0.000341	0.00193	0.00102
3	Estimate	−4.6119	0.00547	0.0009	−0.0133	0.0103
	Standard Error	0.5208	0.000598	0.000334	0.00227	0.00134
4	Estimate	−3.0052	0.00354	0.000574	−0.0120	0.00665
	Standard Error	0.4760	0.000435	0.000474	0.00208	0.00105
5	Estimate	−3.1693	0.00406	0.00203	−0.0163	0.00448
	Standard Error	0.4513	0.00048	0.000444	0.00232	0.00114
6	Estimate	−3.2954	0.00352	0.00142	−0.00882	0.00648
	Standard Error	0.4449	0.000437	0.000411	0.00187	0.00104
7	Estimate	−3.3723	0.00404	0.000985	−0.00866	0.00694
	Standard Error	0.4554	0.000485	0.000339	0.00199	0.00113
8	Estimate	−3.638	0.00369	0.0012	−0.0112	0.0078
	Standard Error	0.4341	0.000442	0.000337	0.00193	0.00103
9	Estimate	−4.1649	0.00425	0.00143	−0.0115	0.00796
	Standard Error	0.4762	0.000474	0.000377	0.00202	0.00106
10	Estimate	−3.7145	0.00374	0.00108	−0.0117	0.00813
	Standard Error	0.4397	0.000442	0.000321	0.00202	0.00104
11	Estimate	−3.9929	0.00394	0.00135	−0.0116	0.00834
	Standard Error	0.4634	0.000456	0.000363	0.00201	0.00107
12	Estimate	−4.4621	0.00453	0.00133	−0.011	0.00823
	Standard Error	0.4935	0.0005	0.000362	0.002	0.00108
13	Estimate	−3.256	0.00343	0.00129	−0.0121	0.0077
	Standard Error	0.4408	0.00044	0.000359	0.00197	0.00103
14	Estimate	−3.1481	0.00333	0.00132	−0.0124	0.00767
	Standard Error	0.443	0.000442	0.000366	0.00199	0.00102
15	Estimate	−3.3662	0.00354	0.00121	−0.0116	0.00763
	Standard Error	0.4402	0.000445	0.000343	0.00193	0.00102

**Table 10:** The confusion matrix of the method that includes all four hormone metabolites assayed with the predictive value.

Confusion Matrix (TES, EST, PRO and COR)					
	Observed		Forecast		
			SEX		Correct Percentage
			1	0	
Step 1	SEX	1	228 (40.57%)	72 (12.81%)	76.00
		0	43 (7.65%)	219 (38.97%)	83.59
	Overall percentage				**79.54**

**Table 11:** The confusion matrix of the method that includes different groups of the hormone metabolites assayed (TES and EST vs. TES, EST and PRO) with the predictive value.

**Confusion Matrix (TES, EST)**					
	**Observed**		**Forecast**		
			**SEX**		**Correct Percentage**
			**1**	**0**	
Step 1	SEX	1	221 (39.32%)	79 (13.88%)	73.67
		0	91 (16.19%)	171 (30.43%)	65.27
	Overall percentage				**69.75**
**Confusion Matrix (TES, EST and PRO)**					
	**Observed**		**Forecast**		
			**SEX**		**Correct Percentage**
			**1**	**0**	
Step 1	SEX	1	212 (37.72%)	88 (15.66%)	70.67
		0	88 (15.66%)	174 (30.96%)	66.41
	Overall percentage				**68.68**

## Data Availability

The data that support the findings of this study are available from the corresponding author upon reasonable request.
